# Facile mutant identification via a single parental backcross method and application of whole genome sequencing based mapping pipelines

**DOI:** 10.3389/fpls.2013.00362

**Published:** 2013-09-13

**Authors:** Robert S. Allen, Kenlee Nakasugi, Rachel L. Doran, Anthony A. Millar, Peter M. Waterhouse

**Affiliations:** ^1^School of Molecular Bioscience, University of SydneySydney, NSW, Australia; ^2^School of Biological Sciences, University of SydneySydney, NSW, Australia; ^3^Plant Sciences Division, Research School of Biology, Australian National UniversityCanberra, ACT, Australia

**Keywords:** Arabidopsis, mutant-mapping, NextGen-Sequencing, parental-backcross, EMS, mutant-screen

## Abstract

Forward genetic screens have identified numerous genes involved in development and metabolism, and remain a cornerstone of biological research. However, to locate a causal mutation, the practice of crossing to a polymorphic background to generate a mapping population can be problematic if the mutant phenotype is difficult to recognize in the hybrid F2 progeny, or dependent on parental specific traits. Here in a screen for leaf hyponasty mutants, we have performed a single backcross of an Ethane Methyl Sulphonate (EMS) generated hyponastic mutant to its parent. Whole genome deep sequencing of a bulked homozygous F2 population and analysis via the Next Generation EMS mutation mapping pipeline (NGM) unambiguously determined the causal mutation to be a single nucleotide polymorphisim (SNP) residing in *HASTY*, a previously characterized gene involved in microRNA biogenesis. We have evaluated the feasibility of this backcross approach using three additional SNP mapping pipelines; SHOREmap, the GATK pipeline, and the samtools pipeline. Although there was variance in the identification of EMS SNPs, all returned the same outcome in clearly identifying the causal mutation in *HASTY.* The simplicity of performing a single parental backcross and genome sequencing a small pool of segregating mutants has great promise for identifying mutations that may be difficult to map using conventional approaches.

## Introduction

A forward genetic approach has been the foundation of determining gene function for many decades. However, map-based cloning has historically been a labor intensive and cumbersome process, often involving outcrossing of the mutant to a polymorphic line followed by fine mapping using a pool of potentially thousands of individual F2 plants carrying the mutation (Jander et al., 2002). Outcrossing can also be problematic in screens that are reliant on multiple mutations, where each additional recessive mutation quadruples the number of F2s plants required to obtain homozygous mutants. Additionally, as many traits that occur in certain ecotypes or cultivars of plants are altered when crossed to others, their accurate phenotyping in F2 polymorphic backgrounds can be challenging (Page and Grossniklaus, 2002; Mallory et al., 2009).

Recent advances in deep sequencing have revolutionised the identification of causal mutations underlying a particular mutant phenotype. The ability to rapidly sequence plant genomes has greatly facilitated the identification of mutants using the principle of bulk segregant analysis (Michelmore et al., 1991), where DNA from tens to thousands of individual segregants may be whole genome sequenced simultaneously (Schneeberger et al., 2009b). An underlying tenet relies on evaluating the frequency and position of mutation induced single nucleotide polymorphisms (SNPs) in the pool of mutant F2 individuals. Theoretically, the recessive causal mutation will be always homozygous, whereas the homozygosity of linked SNPs will decrease with distance from the causal mutation. Thus, the position of these linked SNPs, and the measure of their homozygosity/allelic frequency can be used as markers to identify the causal mutation with strong likelihood.

Using this principle, two independent groups have developed web-accessible computational software to map causative mutations; Next-Generation EMS mutation mapping (NGM, Austin et al., 2011) and SHOREmap (Schneeberger et al., 2009b). The first demonstrations of the methods were in Arabidopsis, where Ethane Methyl Sulphonate (EMS) mutations were mapped using polymorphic Landsberg *erecta* (L*er*) × Columbia (Col-0) bulk F2 segregants. The utility of the SHOREmap pipeline has extended to include mapping of non-EMS mutations in non-model plants (Guo et al., 2012). In both methods, regions that were scarce for polymorphic L*er* SNPs were firstly identified as potentially harboring the mutation, and then these were scanned for EMS SNP frequency. Although both of these methods take similar approaches, NGM was demonstrated to have greater success in identifying mutation causative SNPs using a smaller number of F2 individuals (Austin et al., 2011). However, it is unclear exactly what feature(s) of the NGM pipeline provided an advantage for mutant identification with fewer individuals.

Recently, further studies have circumvented outcrossing by using a backcross to parent method (Abe et al., 2012; Hartwig et al., 2012). The method employed by Abe et al. (2012) was significant in demonstrating the ability to rapidly map agronomically important rice traits that may not have been revealed by outcrossing to a polymorphic background, as the subtle traits may have been masked by genetic variation. However, the initial bulk segregant analysis was unable to distinguish among several candidates and further transgenic analysis was required to unambiguously determine the causative mutation. The method employed by Hartwig et al. (2012) also used the backcross principle combined with elements of the SHORE pipeline for mapping analysis (SHOREbackcross), and although further targeted deep sequencing of candidates was required to discriminate the causative SNP from other closely linked SNPs (Hartwig et al., 2012), this was a clear demonstration of the capability of the method. More recently it has been demonstrated that mapping by sequencing can be carried out by direct sequencing of individual allelic mutant genomes (Nordstrom et al., 2013). However, it was also demonstrated via *in-silico* modeling that background mutations can render unambiguous casual mutant identification more difficult using this principle than if a bulk-segregant population is utilized (James et al., 2013).

We have performed a sensitized forward genetic screen in an attempt to isolate mutants involved in the Arabidopsis microRNA (miRNA) pathway. To do this we used a loss-of-function *mir159a* T-DNA mutant as the parent for EMS mutagenesis. In Arabidopsis, miR159 is predominantly encoded by two functionally redundant genes, *MIR159a* and *MIR159b*. Although mutation of *MIR159a* reduces total miR159 levels to approximately 10% of wild-type, *mir159a* plants are morphologically indistinguishable from wild-type. However, when total miR159 levels are reduced further in a *mir159ab* double mutant, a distinctive phenotype characterized by upward curling leaves (hyponasty) is observed, due to the deregulation of miR159 target genes *MYB33* and *MYB65* (Allen et al., 2007, 2010). Thus, in the *mir159a* mutant, subtle perturbation of miRNA activity may result in a morphological outcome that would not be manifested in wild-type plants. Further, leaf hyponasty is a phenotype often associated with loss-of-function in general miRNA biogenesis componentry. Therefore, by screening for hyponastic mutants using the *mir159a* background, we aimed to find mutants either specifically involved in miR159 biogenesis/efficacy or function, or involved in the general miRNA pathway that may not be apparent in wild-type plants. Here, we have identified a causative SNP from a mutant obtained from this screen. We demonstrate that the parental backcross method, combined with the NGM pipeline that was originally designed for the outcross method, can be utilized to unambiguously map an Arabidopsis mutant from a small pool of F2s with relatively low sequence coverage and without resorting to successive rounds of deep sequencing. This rapid and facile method has great promise to economically identify Arabidopsis mutants that may be difficult to map using more conventional approaches.

## Results

### Selection of a mutant potentially involved in the miRNA pathway

**Figure 1:**
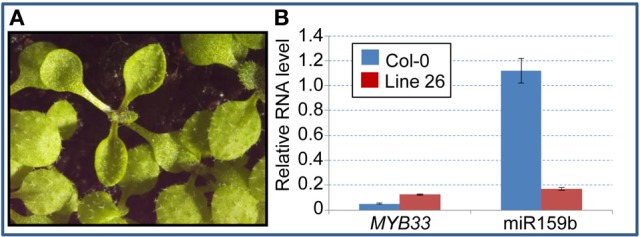
**Phenotype and molecular characteristics of the Line 26 hyponastic mutant. (A)** A Line 26 M2 hyponastic mutant shown adjacent to wild type M2 siblings. **(B)** qRT-PCR analysis of *MYB33* and mature miR159b expression in the Line 26 hyponastic mutant. Analysis was performed on RNA extracted from 3-week-old plants and mRNA abundance was normalized to cyclophilin, and miRNA analysis was normalised to *sno101*, measurements being the average of three replicates with error bars representing the standard error of the mean.

After EMS treatment of *mir159a* seed, 500 M1 plants were self-pollinated, and ~50 M2 plants from each original M1 plant were grown; thus ~25 000 M2 plants were screened for leaf hyponasty. From this initial screen, twenty hyponastic M2 lines were identified. Of these, line 26 was chosen for further analysis due to its phenotypic similarity to miRNA biogenesis mutants (Figure 1A). As a secondary screen to determine if the line 26 mutant was involved in miRNA biogenesis and/or function, we performed qRT-PCR on the miR159 target gene *MYB33*, which is known to be elevated in *mir159ab* and certain other miRNA biogenesis mutants (Han et al., 2004; Park et al., 2005; Allen et al., 2007). We also assayed for mature miR159, which would be expected to be lower if the mutant was negatively affected in miRNA biogenesis. We found *MYB33* levels were considerably higher than wild-type, and mature miR159b levels were also substantially lower (Figure 1B). Together with the phenotype of this mutant, the data indicated that the line 26 mutant was a strong candidate for involvement in miRNA biogenesis.

### Generation of a parental backcross F2 mapping population and whole genome next-generation sequencing

**Figure 2:**
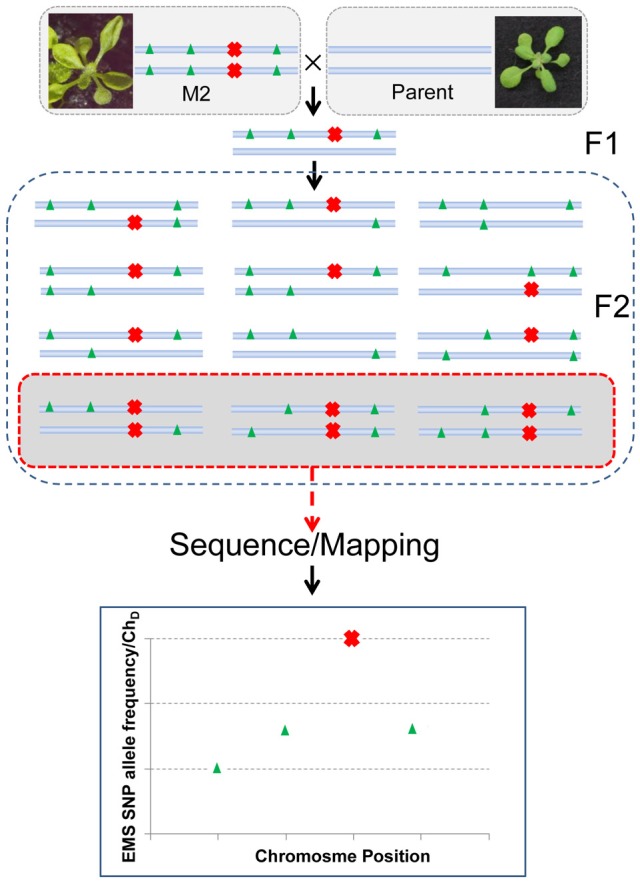
**The backcross to parent principle for identifying the causative EMS generated SNP**. The M2 mutant is crossed with the parent and allowed to self-pollinate. If the mutation (red cross) is recessive the F2 progeny will segregate for the mutation in a 1:3 ratio. Individuals showing the mutation are pooled and a bulk DNA prep is sequenced. Because the mutation has been selected for the causal SNP should be homozygous in all reads. This can be represented as the allele frequency or the dischordant chastity statistic (Ch_D_) if using NGM software. Linked EMS mutations (green triangles) will have a SNP allelic frequency approaching 1 the closer they are to the causative mutation. Resolving closely linked mutations will be dependent on their distance to the causal mutation, the sequencing coverage, and the number of F2s used. The simplified scheme shown assumes no differences between the parent and reference genome. Sequencing the parent genome is required if differences are expected between the reference genome and the parental strain used.

As the phenotypes of some miRNA mutants are not always apparent in different backgrounds (Mallory et al., 2009), we wanted to avoid crossing the mutant to a polymorphic background. Therefore, we crossed the mutant back to its parent (*mir159a*), and aimed to use the EMS generated SNPs rather than ecotype specific SNPs as markers to map the mutation. The principle of the method is shown in Figure 2. After a single backcross, 110 progeny (referred to as BCF_2_) showing the hyponastic phenotype were selected from a segregating pool (340 wild type:110 mutant, consistent with a single recessive causal mutation) of F2 individuals. The plant material of these individuals was pooled and the DNA was extracted from both the BCF_2_ pool and also from the *mir159a* parent for deep sequencing. As laboratory strains have been shown to contain up to several thousand SNP differences compared to the Col-0 reference genome (Uchida et al., 2011), we also sequenced the parent, to enable filtering out of any non EMS SNPs differences between our laboratory grown *mir159a* Col-0 background and the Col-0 reference genome. Deep sequencing yielded a total of 31,786,144 paired-end reads for the parent and 33,726,432 paired-end reads for the mutant pool (both 100 nt reads). Pre-processing of reads by the trimmomatic software (Lohse et al., 2012) resulted in the vast majority of reads being retained for both, including an average read length of 99 nts (Supplementary Table 1). Of these, 29,438,383 and 30,644,168 properly paired reads from parent and mutant, respectively, could be mapped back to the TAIR9 genome. This gave an average total genomic coverage of 48 and 50 times for the parent and mutant pool (BCF_2_), respectively.

### Mutant identification using next generation mapping (NGM)

The utility of NGM software in identifying causal SNPs has been demonstrated using mapping populations derived from a cross between EMS mutated Col-0 and polymorphic L*er* (Austin et al., 2011). NGM offers a simple user friendly interface that is suitable for non-specialists, making it broadly useful for plant genetic studies. We reasoned that this pipeline could also be used for identifying and assessing EMS SNPs of the mutant F2 bulk DNA derived from a parental backcross. The first step in the default NGM pipeline aligns the SNPs derived from the sequencing of pooled DNA of bulk F2 (Col-0 × L*er*) segregants against the Col-0 reference genome. This identifies the “SNP desert”—a region reduced in SNPs derived from the polymorphic ecotype used in the cross. The L*er* specific SNPs should be reduced in this SNP desert due to selection for the Col-0 derived mutation. Obviously this would not be the case if the mutant was back-crossed to the parent; as both the mutant and parent are of the same ecotype, only EMS SNPs should be present, where the SNPs that are linked to the mutation would have an allelic frequency affected by the rate of recombination in the region, and the strength of phenotypic selection, producing a SNP peak.

To identify and assess these EMS SNPs, we used the NGM web-portal using the “discordant chastity statistic” (Ch_D_). This is a function of the “purity” of a SNP at a certain position, and is calculated by dividing the number of observations of the most-frequent non-reference base by the sum of the abundances of the two most common bases (Austin et al., 2011). Therefore, if all reads displayed a single non-reference base, then the discordant chastity would be 1 (i.e., a homozygous state). Because the ratio of homozygous to heterozygous EMS SNPs would be expected to increase with proximity to the mutation, we expected to find SNPs with a Ch_D_ approaching 1 near our causative mutation. Of note, this NGM method differs to other software such as SHOREmap for calculating allelic frequencies, where the number of non-reference bases as a fraction of the total number of reads covering the position is used.

From the raw sequence data, we used samtools (v0.1.16) pileup (Li et al., 2009) to call SNPs for both the *mir159a* parent and BCF_2_. We then filtered these parental specific SNPs from BCF_2_ (i.e., differences between the *mir159a* parent and the Col-0 reference genome) to compile a modified “.emap” (Austin et al., 2011) file that could be interrogated through the NGM portal. By default the NGM pipeline takes the samtools pileup output to filter out insertions and deletions, and extracts only SNP information, in addition to calculating discordant chastity values per SNP position. This file is then formatted to an “.emap” file for upload onto the NGM web-portal. Therefore, by generating an “.emap” file devoid of parental specific SNPs, we could better interpret the final output from the NGM web-portal. The NGM web portal also by default filters out SNPs that fall outside genic regions.

**Figure 3:**
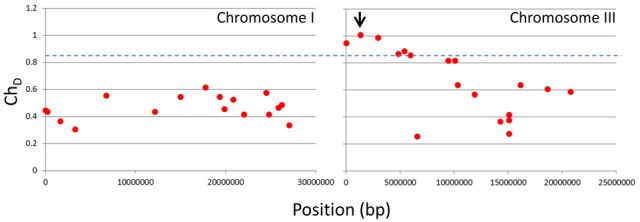
**NGM outputs for chromosomes I and III plotted against genome position**. Parental SNP filtered .emap files were interrogated via the NGM web portal, with the Ch_D_ threshold set at zero, and all SNPs identified are shown as red dots for chromosomes I and III. The blue dashed line indicates the default Ch_D_ threshold of 0.85 used to identify the six SNPs indicated in Table 1. The black arrow indicates the SNP identified in *HASTY*.

Firstly, using the NGM portal with the Ch_D_ threshold set at zero we identified 63 EMS genic SNPs across all five chromosomes. We found no SNPs above the NGM web portals default Ch_D_ threshold of 0.85 on any chromosome except chromosome 3; here the NGM portal identified six SNPs with a Ch_D_ at or above 0.85 (Figure 3). Of these, two were exonic, and only one had a chastity statistic of 1; a stop-gain in *HASTY* (At3g05040) (Table 1). *HASTY* is a well characterized gene involved in miRNA biogenesis and our mutant displayed molecular and phenotypic characteristics (Figure 1) highly similar to previously described *hasty* loss-of-function mutants (Telfer and Poethig, 1998). From these observations, combined with the fact that the only SNP identified with a Ch_D_ of 1 was a stop gain in *HASTY*, it could be established that this SNP, distinctly resolved by NGM, was most likely the causative mutation for line 26.

**Table 1:** **NGM annotation for parental SNP filtered .emap file**.

**Chrom**	**Position**	**Ref base**	**SNP base**	**Depth**	**Ch_D_**	**Accession**	**Position**	**Ref codon**	**SNP codon**	**AA change**
3	82825	C	T	52	0.94	AT3G01270.1	3′UTR			
3	1405085	C	T	31	1	AT3G05040.1	CDS	TGG	TGA	W → ^*^
3	3057628	C	T	45	0.98	AT3G09940.1	CDS	GAG	AAG	E → K
3	4919240	C	Y	36	0.86	AT3G14630.1	CDS	CCG	CYG	P →
3	5482374	C	Y	42	0.88	AT3G16180.1	CDS	AGG	AGY	R →
3	6035523	C	Y	47	0.85	AT3G17650.1	CDS	CTC	CTY	L →

Outputs are shown at the default Ch_D_ threshold of 0.85. For AA changes,

* indicates a stop-gain and blank spaces are indicated when the SNP is not resolved as a discrete nucleotide.

### Different SNP callers produce highly similar outcomes despite differences in SNP estimation

**Figure 4:**
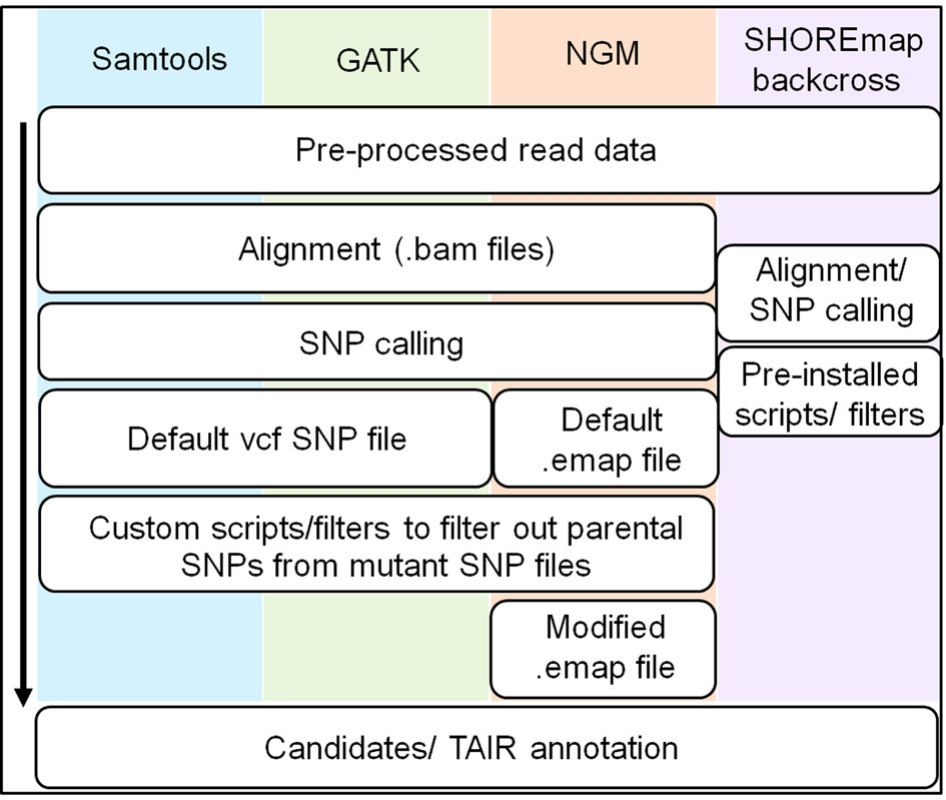
**Overview of workflow for different SNP calling pipelines**. The commonalities and differences between the four SNP calling pipelines are illustrated with the black arrow indicating the direction of the workflow. A detailed overview encompassing specific commands is provided in Supplementary Figure 1. All pipelines generate a SNP file for both parent and mutant lines, after which scripts and filters were used to generate a SNP file for the mutant line lacking parental specific SNPs.

In addition to NGM, there are several programs that can interrogate deep sequencing data for mapping purposes (Li et al., 2009; Schneeberger et al., 2009a; McKenna et al., 2010). Two of these, samtools and the Genome Analysis Toolkit (GATK), are widely used SNP callers employed in many applications, while another called SHOREmap has been developed with built-in utilities that facilitate the identification of a causal mutation (Figure 4). We wanted to determine if the backcross method was sufficiently robust to detect *HASTY* using these other pipelines, and also to assess if any offered a particular advantage for causal SNP determination using the backcross method. Therefore, using separate analyses, SNPs were called using GATK, samtools and SHOREmap algorithms for both the *mir159a* parent and BCF_2_ sequence data.

Both samtools and GATK being “generic” SNP callers, are reasonably flexible in allowing several filters to be manually adjusted. Samtools, by default, implements a Base Alignment Quality (BAQ) concept which attempts to identify false SNPs caused by nearby indels (Li, 2011). However, because this filter could potentially lead to loss of “real” SNPs, we used samtools without the BAQ option (Supplementary Figure 1 and Supplementary Table 2). GATK also uses a base quality score recalibration step to assess the probability of a mismatch against the reference genome, although this requires a database of known polymorphisms to identify legitimate SNPs, which we generated (DePristo et al., 2011). Although GATK was designed for human data, it can be applied to other organisms where known SNPs are not readily available. In our case, an initial round of SNP calling was performed on the parent and BCF_2_ datasets by the Unified Genotyper tool in the GATK pipeline, and these calls were further refined by performing two more rounds of SNP calling by the pipeline (Supplementary Figure 1 and Supplementary Table 2). For both samtools and GATK, parental SNPs were filtered from the BCF_2_ (mutant) data. SHOREmap utilises its own SNP caller and can implement a number of filters including quality scores, read depth and allele frequency, and can filter out parental SNPs. For this study we set the default parameters as used by Hartwig et al. (2012) (Supplementary Figure 1 and Supplementary Table 2).

**Figure 5:**
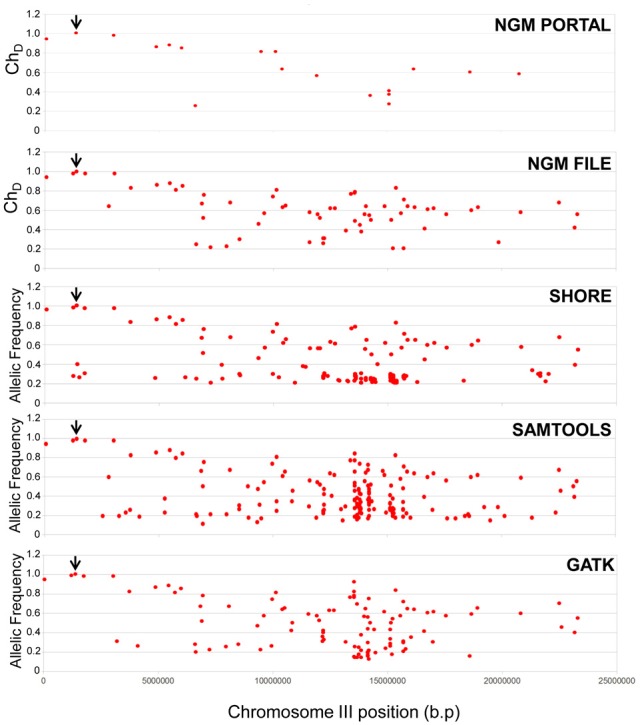
**Comparison of different SNP calling pipelines for causal mutant identification**. Allelic frequencies (GATK/samtools/SHOREmap) or dischordant chastity (NGM) scores for all SNP resolved using *mir159a* parent and BCF_2_ mutant whole genome sequence data. “NGM portal” refers to the web based output where the SNP parent filtered .emap file was interrogated, whereas “NGM file” refers to the analysis of the raw .emap files prior to loading into the web portal, where the mutant line has been filtered of the *mir159a* parent SNPs. The SNP representing the causal mutation in *HASTY* is indicated by the black arrow.

For all pipelines, the only EMS SNP identified as homozygous (i.e., with an allelic frequency of 1) was the same stop-gain SNP in *HASTY* identified previously via NGM (Figure 5). Thus, the backcross principle is sufficiently robust that generic SNP calling programs not specifically tailored for backcross mapping can be employed to correctly identify mutation causal EMS SNPs. While NGM and SHOREmap pipelines allowed a more streamlined workflow for the identification of the causal SNP (for example via plots and calculated mutant allele frequencies), an analysis based simply on parent SNP filtering and calculation of mutant allele frequencies based on read depth from samtools mpileup and GATK was sufficient for the identification of the causal mutation from these pipelines.

Across the alternative pipelines, there was strong concordance between SNPs identified of high mutant allelic frequency (Figure 5 and Supplementary Table 3). Conversely, SNPs that were not identified across all four platforms, and especially those uniquely identified by a particular pipeline, generally had lower allelic frequencies. These outcomes are what would be expected when considering all pipelines should be generally capable of identifying SNPs that are strongly homozygous and of high quality, whereas differences in filtering and quality cut-offs between programs are likely to be reflected in a different range of identified low allelic frequency SNPs. In summary, despite different outcomes from these pipelines in the range and number of SNPs called, those that would be considered important for causal mutant identification were still readily and consistently identified using any of these pipelines.

A further outcome of the SHOREmap, GATK and samtools analysis was that a much higher number of EMS SNPs were identified using these methods than the NGM web portal. SHOREmap, GATK and samtools, identified 515, 391 and 483 EMS SNPs, respectively, and this is in accordance with the expected amount of SNPs produced for such an EMS dose as used in this experiment. The NGM web-portal reports a much lower number of EMS SNPs to a large extent by virtue of the fact that intergenic SNPs are ignored. This is evidenced by the fact that The NGM portal also did not report two SNPs of high allelic frequency within 1.3 and 0.34 Mb of *HASTY* that were identified by all other pipelines (Figure 5 and Supplementary Table 3).

To asses this further, we examined the parent filtered BCF_2_ .emap file, prior to interrogation by the NGM web portal. Here we found a much greater concordance in SNP frequency with the other three platforms (Figure 5), where the number of SNPs identified (that would include intergenic SNPs) was 408. Therefore, in comparison to these other methods, the NGM focus on SNPs of potentially greater relevance would obviously hinder mapping of intergenic casual mutations. Nevertheless, despite these apparently major differences in SNP calling and filtering between NGM and the other software, importantly all four pipelines converge on the same result in clearly isolating the mutation causative SNP.

Of note, we did not carry out PCR duplicate removal from our reads alignment data prior to our SNP calling, apart from the GATK pipeline which does this by default. We had initially found 9.2 and 6.3% of the reads to be duplicates (based on the Picard software “MarkDuplicates” utility), in our parent and mutant, respectively, but did not find any difference in results whether we removed duplicates or not. Nonetheless, it is still good practice to remove duplicates prior to SNP calling and would recommend users to assess the effect of PCR duplicate removal from their reads data.

## Discussion

We have identified an EMS generated mutant using solely the position and frequency of EMS SNPs determined by whole genome sequencing. To our knowledge this is the first report of using a parental backcross method to unambiguously pinpoint a causal mutant without the requirement for additional approaches, such as further targeted sequencing or transgenic methods. Although we have only utilized this method to identify a single causative SNP, our approach was facile in accomplishing a task previously considered cumbersome and labor intensive. It was remarkable that the NGM pipeline, although originally designed to map genes using hybrid mapping populations of Col-0 × L*er*, was not only able to narrow down the causal candidates to a single gene, but also that so few candidates were actually identified. Previous examples in the literature failed to identify the causal mutation after the first round of deep sequencing of bulk segregant pools (Ashelford et al., 2011; Hartwig et al., 2012). Therefore, our example demonstrates that the backcross method can potentially identify mutants even more easily than previously reported.

There are several variables that may have contributed to the ease with which our mutant was identified. Firstly if the EMS load was relatively low, or unevenly distributed across the *mir159a* genome, it might be expected that it would be easier to identify the causal mutation simply because there would be fewer candidates. However, it could also be argued that more EMS SNPs may improve the resolution of the SNP distribution peak and thus the ability to identify the causal mutation. In any case, it appears the EMS load of 515 SNPs (as determined by the SHOREmap method) for the mutant, does not appear to be greatly less than other reports (Ashelford et al., 2011; Hartwig et al., 2012), and therefore this is not likely to be a major factor in the relative ease of the causal SNP identification. Another factor that could account for the ease in identifying the SNP in *HASTY* was the level of recombination- a high level between the causal SNP and a closely linked non-causal SNP would allow discrimination between the two, provided a sufficient number of recombinants were chosen. Recently, a report that examined levels of recombination in various Arabidopsis F2 populations found large variances in recombination frequencies among different populations (Salome et al., 2012), and while the 1 Mb region immediately surrounding *HASTY* appears to be on average an area of generally low recombination, there is considerably more average variation in the ~5 Mb region that encompasses the linked SNPs identified by both NGM and SHOREmap/GATK (Salome et al., 2011). We may have been fortunate that our mutation was in this area, and the fact that the linked SNPs still had very high allelic frequencies or discordant chastises suggests we were very close to the limit of resolving *HASTY* from the other linked EMS SNPs. Based on this result, a precautionary principle for future studies would to be to maximize both the number of recombinant F2s and the coverage to enable discrimination between SNPs, particularly for cases where the mutation may reside in a region of low recombination. Indeed a recent large scale simulation of mapping by sequencing (James et al., 2013) took advantage of the experimentally derived recombination landscape described above (Salome et al., 2012); here it was found that because of the lower levels of available markers used in backcross mapping (EMS SNPs as opposed to ecotype specific SNPs), coverage of ~50×, and 50 pooled individuals was suited for identification of candidate mutants. The fact our experimental conditions met or exceeded these parameters (average 50× coverage and 110 pooled individuals) further validates these recommendations. However, as demonstrated by Hartwig et al. (2012), even “conventional” deep sequencing may not be able to discriminate closely linked mutations, and in such instances targeted ultra-deep sequencing of candidate regions may still be required.

This ease of identification did not appear to be due to a particular ability of the NGM pipeline's unique calculation of SNP homozygosity, as all other pipelines we tested returned the same net result and were able to discriminate the causative mutation from other closely linked SNPs. Nevertheless, there were several differences in the output from the pipelines that relates to their intrinsic filters where the quality scores of a particular SNP can have a different value and meaning from different software. GATK for example implements a base quality score recalibration step, which takes into account a number of properties such as mismatches due to close-by indels, sequence context (e.g., dinucleotide content near the SNP), and position of the SNP along the read. This relies on a database of known SNPs, which had to be generated *ad hoc* for the pipeline implemented in this report, as GATK was designed for human data (DePristo et al., 2011). Samtools on the other hand implements a different model to take into account indels that induce mismatching, generating false SNP calls (Base Alignment Quality method) (Li et al., 2009), and is less rigorous than the GATK pipeline. However, these implementations also depend on the initial data quality and depth, which can vary between samples even in the same experiment.

The other pipelines used in this study also possess different procedures and filters. The result is that while some of these can be controlled, this usually incurs flow on effects to downstream parts of a pipeline and will generate different sets of SNPs between the programs. Filters should therefore be empirically tested. However, despite the SNP calling intricacies, in practice if the correct methods and filters have been implemented, high quality SNPs should still be called, as demonstrated in this study. An important caveat is that while we could identify the causative mutation regardless of the platform used, other mutant screens that may potentially be compromised by less recombination in the causative mutation region, lower mutational loads or weaker selection may be harder to identify using a particular pipeline. Overall, we believe it may be beneficial to implement a variety of methods based on the same input data (as done in this study).

Finally we were fortunate in selecting a phenotype that was simple to isolate from wild type segregants. The fact that only the SNP in *HASTY* had a discordant chastity/allelic frequency of 1 demonstrates the selection was specific and possibly all 110 F2s selected for the bulk segregant pool were homozygous for the causative mutation. This is important because due to the very high discordant chastises/allelic frequencies we found for linked SNPs, contamination by a single wild type segregant may have blurred the distinction between causal and non-causal but closely linked SNPs. This has recently been also demonstrated *in-silico*, where assessment of mis-scoring revealed drastic effects on the resolution of mapping by sequencing outcomes (James et al., 2013). Of more general relevance, although the *hasty* phenotype is not masked in L*er* plants, this ecotype can also produce mildly hyponastic leaves that could have theoretically led to wild-type contamination if a classical Col-0 × L*er* F2 population was used for mapping this particular mutation. This risk is completely circumvented here using the parental backcross method, where any phenotype distortion produced by the introduction of polymorphic backgrounds is completely removed. Lastly, the concept of crossing back to a parent allows for the retainment of parental specific traits such as multiple T-DNA alleles that may be required for the mutation of interest in enhancer or modifier screens. We envisage this method will greatly facilitate screens in such complex genetic backgrounds that would be traditionally hard to undertake if reliant on outcrossing to polymorphic backgrounds.

## Methods

### EMS mutagenesis and growth of arabidopsis

Approximately 5000 seed of the *mir159a* mutant (Allen et al., 2007) were immersed in 0.025% ethylmethanesulfonate (Sigma) overnight with gentle agitation. Approximately 500 EMS treated seeds were planted in soil and grown under 16 h light/8 h dark at 22°C. 500 M1 plants were grown and allowed to self-pollinate, and approximately 50 M2 seeds from each original M1 plant were grown in soil under the same conditions. In this way ~25000 plants were screened for the presence of leaf hyponasty.

### Identification of mutants and construction of a mapping population

A leaf curl mutant M2 line (Line 26) was identified and pollen from the *mir159a* parent was crossed onto Line 26. The F_1_ progeny of this cross was allowed to self-pollinate and a population of F2 individuals (referred to as BCF_2_) was grown to identify mutants. One hundred and ten individuals showing the leaf hyponasty phenotype were selected from the BCF_2_ population. A small leaf from each plant was used for a pooled DNA extraction of all 110 individuals. A Qiagen Plant DNA maxi-kit was used for Arabidopsis genomic DNA extraction.

### Gene expression analysis

RNA from was extracted from a pool of ten 3 week old plants for Line 26 and Col-0 using Trizol reagent. Real time PCR for *MYB33* and mir159b was performed as described previously (Allen et al., 2010).

### Deep sequencing

Libraries of pooled DNA were prepared for deep sequencing using a Qiagen plant maxi kit and the Illumina Truseq genomic sample preparation and multiplex protocol, and sequenced on the Illumina HiSeq2000 sequencer according to the manufacturer's instructions at the Australian Genome Research Facility as paired-end 100 nt reads. Data was outputted as Sanger-format fastq reads. The raw sequencing data can be retrieved from the Short Read Archive at NCBI, under accessions SAMN02324419 and SAMN02324420.

### Bioinformatics

The quality of the deep sequencing output was assessed using the FastQC software (http://www.bioinformatics.babraham.ac.uk/projects/fastqc/), and was trimmed and quality filtered with the Trimmomatic software (Lohse et al., 2012). Reads were aligned to the *A. thaliana* (TAIR9) genome with BWA sampe (Li and Durbin, 2009). The resultant alignment files were converted to sorted bam files using the samtools v0.1.18 package (Li et al., 2009), and were used as input for the subsequent SNP calling analyses.

SNPs were called using four software packages, on both the parent and mutant line. For samtools v0.1.18 (Li et al., 2009) the mpileup tool was used with the following parameters: −Buq, −C50, −Q30. This was followed by filtering with bcftools SNP calling, and filtering with vcftools varFilter setting only “−Q0,” along with other default parameters. For the genome analysis toolkit v2 (GATK) (McKenna et al., 2010; DePristo et al., 2011), the “Best Practice Variant Detection v4” pipeline was applied, with three rounds of “Unified Genotyper” SNP calling, according to the developers recommendations on processing non-human genomes (http://www.broadinstitute.org/gatk/guide/article?id=1247). The output of each round of SNP calling was used as the input for the next round of base score recalibration and unified genotyper calling. For the NGM pipeline (Austin et al., 2011), as per the developers instructions, samtools (v0.1.16) pileup was applied as the SNP caller. The SNP calls were then further processed into .emap files using a conversion script provided on the NGM website. The .emap files were then processed and visualized with the NGM software via a web-portal to assess SNPs with associated discordant chastity values. Parent SNPs-filtered, mutant specific .emap files were also generated by uncompressing the .emap files and using standard bash commands to identify mutant specific SNPs. For SHOREmap (Schneeberger et al., 2009b; Hartwig et al., 2012), the SHORE software was used to align the reads (implementing BWA) and call SNPs according to the instructions in Hartwig et al. (2012). SHOREmap backcross was then implemented to calculate mutant allele frequencies and filter out parent SNPs. Where appropriate, custom scripts were used to identify mutant specific, EMS SNPs and to filter out parent SNPs. ANNOVAR was used to annotate the SNPs (Wang et al., 2010). For the samtools mpileup and GATK pipelines, homozygous SNPs were defined by calculating the mutant allele frequency based on the ratio of mutant allele reads to total reads at a particular locus. For the NGM pipeline, homozygous SNPs were defined based on the discordant chastity metric, and for SHOREmap backcross, the mutant allele frequency (also as the ratio mutant allele reads to total reads) calculated natively by the software was used.

### Conflict of interest statement

The authors declare that the research was conducted in the absence of any commercial or financial relationships that could be construed as a potential conflict of interest.

## Supplementary material

Supplementary Figure S1 EMS SNP calling workflows with relevant commands for NGM/SHOREMAP backcross/samtools and GATK pipelines.

Supplementary Table 1 Read statistics for Illumina Hiseq2000 mapping.

Supplementary Table 2 Commands for SNP caller workflows.

Supplementary Table 3 List of SNPs identified on chromosome III of BCF2 for different SNP calling pipelines.
