# Supplementary figures and images for: Facile mutant identification via a single parental backcross method and application of whole genome sequencing based mapping pipelines
Source: Front Plant Sci. 2013 Sep 13;4:362. doi: 10.3389/fpls.2013.00362 (PMC3772335; doi:10.3389/fpls.2013.00362)

**Supplementary Figure 1**

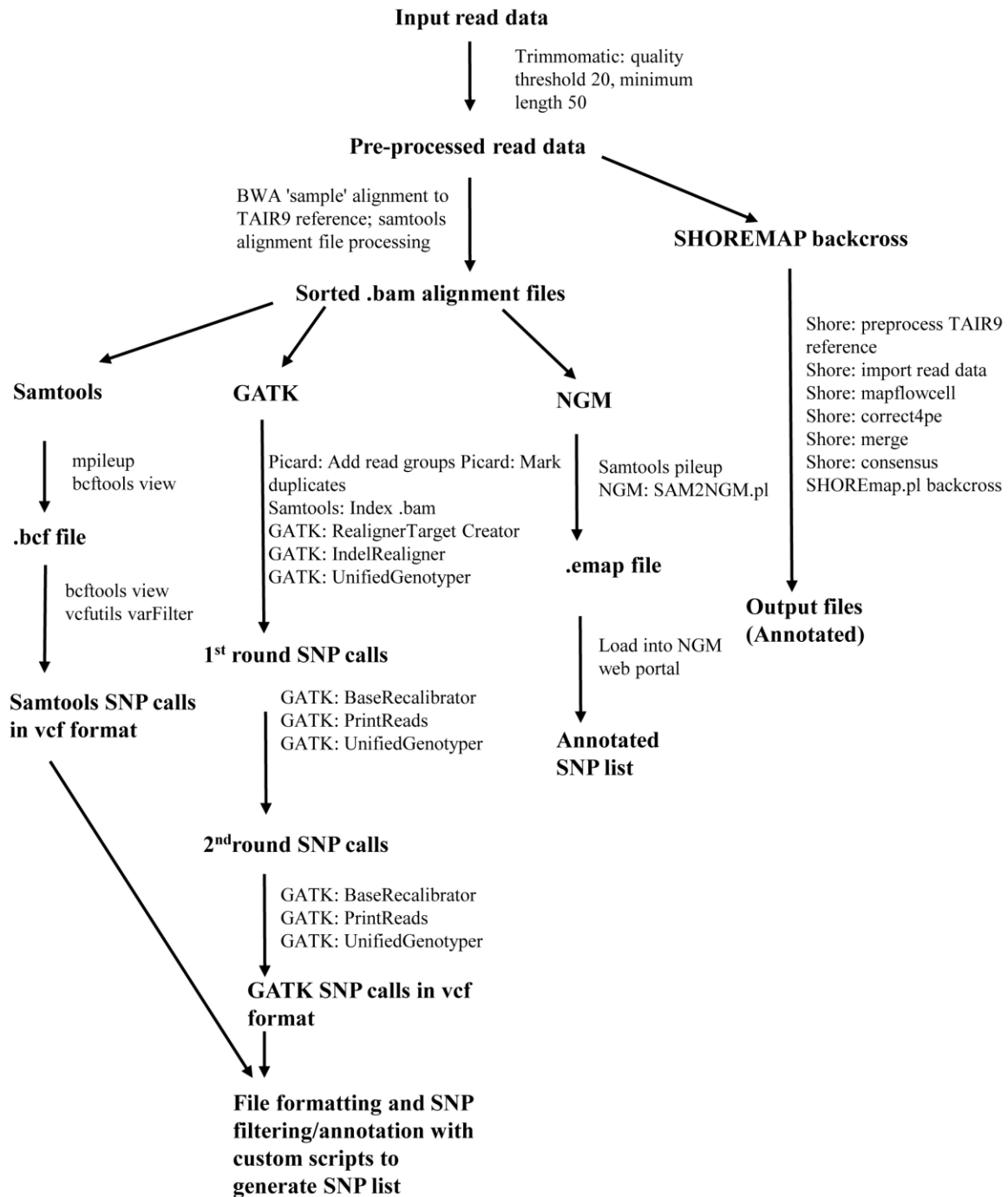

Supplement: Supplementary Figure S1 — EMS SNP calling workflows with relevant commands for NGM/SHOREMAP backcross/samtools and GATK pipelines. [file Presentation1.PDF]
