# Supplementary material for: Facile mutant identification via a single parental backcross method and application of whole genome sequencing based mapping pipelines
Source: Front Plant Sci. 2013 Sep 13;4:362. doi: 10.3389/fpls.2013.00362 (PMC3772335; doi:10.3389/fpls.2013.00362)
Supplement: Supplementary Table 2 — Commands for SNP caller workflows. [file DataSheet2.PDF]

Supplemental table 2: commands for SNP caller workflows

NGM

- 1. Call SNPs
  samtools pileup -cvf TAIR9_ref_file sorted.bam_file > output.pileup
- 2. Generate.emap file
  SAM2NGM.ploutput.pileup

SAMTOOLS

- 1. Prepare input file for SNP calling
  samtools mpileup -C50 -Q30 -Buf TAIR9_ref_file sorted_bam_file | bcftools view -bvcg - > raw.bcf
- 2. Call SNPs
  bcftools view raw.bcf | vcfutils.pl varFilter -Q0 > filtered.vcf

GATK

- 1. Add read groups (Picard tools)

AddOrReplaceReadGroups.jar I=sorted.bam_file O=s1.rg.bam RGLB=genome RGPL=ILLUMINA RGPU=GATKv4 RGSM=sample_name VALIDATION_STRINGENCY=LENIENT

- 2. Mark duplicates (Picard tools)

MarkDuplicates.jar INPUT=s1.rg.bam OUTPUT=s2.dedup.bam ASSUME_SORTED=TRUE VALIDATION_STRINGENCY=LENIENT METRICS_FILE=s2.dedup.metrics

- 3. Index (samtools)

samtools index s2.dedup.bam

- 4. Realign reads (create intervals first, then do IndelRealigner) (GATK)

GenomeAnalysisTK.jar -I s2.dedup.bam -R ref_file -T RealignerTargetCreator -o s3.intervals
GenomeAnalysisTK.jar -T IndelRealigner -I s2.dedup.bam -R ref_file -targetIntervals s3.intervals -o s4.realn.bam

- 5. Unified genotyper (GATK)

GenomeAnalysisTK.jar -T UnifiedGenotyper -R ref_file -I s4.realn.bam -glm BOTH -o s5.UG1.vcf -mbq 30 -nt 4

- 6. Base score recalibrator (GATK)

GenomeAnalysisTK.jar -T BaseRecalibrator -I s4.realn.bam -R ref_file -knownSites s5.UG1.vcf -o s6.recal

- 7. Print Reads (GATK)

GenomeAnalysisTK.jar -T PrintReads -R ref_file -I s4.realn.bam -BQSR s6.recal -o s7.recal.bam

- 8. Unified Genotype (GATK)

GenomeAnalysisTK.jar -T UnifiedGenotyper -R ref_file -I s7.recal.bam -glm BOTH -o s8.UG2.vcf -mbq 30

- 9. Base score recalibrator (GATK)

GenomeAnalysisTK.jar -T BaseRecalibrator -I s4.realn.bam -R ref_file -knownSites s8.UG1.vcf -o s9.recal

- 10. Print Reads (GATK)

GenomeAnalysisTK.jar -T PrintReads -R ref_file -I s4.realn.bam -BQSR s9.recal -o s10.recal.bam

- 11. Unified Genotyper (GATK)

GenomeAnalysisTK.jar -T UnifiedGenotyper -R ref_file -I s10.recal.bam -glm BOTH

SHOREMAP backcross

- 1. Preprocess the reference file

shore preprocess -f TAIR9_ref_file -i TAIR9 -W

- 2. Import reads

shore import -v Fastq -a genomic -x read1.fastq -y read2.fastq --rplot --disable-illumina-filter -k 50 -o sample_name

- 3. Map reads using shore

shore mapflowcell -f sample_name -i TAIR9_ref_file.shore -v bwa -n 10% -g 7% -p -c 2 -b 250000 -M 2 -P replace

- 4. Merge

shore merge -p -m sample_name -o sample_name/merge

- 5. Call SNPs

shore consensus -n sample_name -f TAIR9_ref_file.shore -o sample_name/consensus -i sample_name/merge/map.list.gz -g 4 -a scoring_matrix_het.txt -v -r

- 6. Use SHOREmap.pl backcross (require TAIR9 chr sizes; require steps 1 to 5 on parent line for "--bg" option)

SHOREmap.pl backcross --marker sample_name/consensus/ConsensusAnalysis/quality_variant.txt --out shoremapout --chrsizes TAIR9_chr_sizes.txt --bg parent_name/consensus/ConsensusAnalysis/quality_variant.txt --marker-score 25 --marker-freq 95 --marker-cov 8 --bg-freq 20
